# Effectiveness of Bite-Size Teaching for Mental Health Professionals

**DOI:** 10.1192/bjo.2026.11327

**Published:** 2026-06-30

**Authors:** Nilamadhab Kar, Natalie Cook, Maryam Imran Aziz, Humza Ilyaz

**Affiliations:** 1 Black Country Healthcare NHS Foundation Trust, Wolverhampton, United Kingdom; 2 University of Wolverhampton, Wolverhampton, United Kingdom

## Abstract

**Aims:**

Bitesize teaching is a short session delivered on a specific topic to enhance the knowledge and confidence of practitioners and to support practical application of clinical knowledge. In psychiatry, bite-size teaching has been suggested for training ward staff in physical health-related topics. However, it has been used in a wide range of topics in different teams. We intended to see the effectiveness of bite-size teaching across different professional groups in a range of clinical settings, covering a variety of topics relevant to mental health professionals.

**Methods:**

We compared the change in knowledge of a topic and skill, confidence, and comfort level in performing a related task through a pre- and post-session assessment using the Teaching Effectiveness Questionnaire (TEQ), and open-ended questions about learning. The teaching sessions included different professional groups such as doctors, nurses, and allied health professionals; and covered various topics.

**Results:**

There were 149 attendee responses, over the range of topics covered in the bitesize teaching, e.g. role of exercise, green spaces and mental health, diagnostic formulation, metabolic disorder, suicidality management, dementia prevention, Mental Health Act, community treatment order, etc. in multiple sessions. Most of the attendees were doctors(65.1%), followed by nurses (17.4%), and the rest were allied health professionals. The content of the sessions was reported to be very good in 47.0%, and good in 37.6%; session delivery was observed as very good 50.3%, and good 38.9%. There were positive correlations between years of experience and knowledge, skill, and confidence in performing, comfort level, and teaching the task. Pre and post-teaching scores in these domains also positively correlated. Cronbach's alpha of the TEQ for the sample pre-teaching was 0.97, suggesting an excellent level of internal consistency. There were significant changes pre- and post-teaching session in all the domains, such as knowledge (3.3±1.1 vs. 4.0±0.8), skills (3.2±1.1 vs. 3.9±0.8), confidence (3.2±1.1 vs.4.0±0.8), comfort level (3.3±1.1 vs. 4.0±0.8), confidence in teaching others (3.0±1.1 vs. 3.8±1.0), respectively. The overall change was from 16.0±5.2 to 19.7±4.0 (p<0.001). This was consistent across topics and professional groups. Qualitatively, attendees perceived this method as a quick refresher of existing knowledge, and a source for relevant new information that is useful in clinical practice, especially while supporting patients and families.

**Conclusion:**

It appears that the bitesize teaching is an effective method for training on diverse clinical topics. Post-session responses showed positive changes in domains of knowledge, skills, and confidence. Further studies are required about the persistence of the effectiveness over time.

